# RNA alternative splicing impacts the risk for alcohol use disorder

**DOI:** 10.1038/s41380-023-02111-1

**Published:** 2023-05-23

**Authors:** Rudong Li, Jill L. Reiter, Andy B. Chen, Steven X. Chen, Tatiana Foroud, Howard J. Edenberg, Dongbing Lai, Yunlong Liu

**Affiliations:** 1 Center for Computational Biology and Bioinformatics, Indiana University School of Medicine, Indianapolis, IN 46202 USA; 2 Department of Medical and Molecular Genetics, Indiana University School of Medicine, Indianapolis, IN 46202 USA; 3 Department of Biochemistry and Molecular Biology, Indiana University School of Medicine, Indianapolis, IN 46202 USA

**Keywords:** Genetics, Molecular biology

## Abstract

Alcohol use disorder (AUD) is a complex genetic disorder characterized by problems arising from excessive alcohol consumption. Identifying functional genetic variations that contribute to risk for AUD is a major goal. Alternative splicing of RNA mediates the flow of genetic information from DNA to gene expression and expands proteome diversity. We asked whether alternative splicing could be a risk factor for AUD. Herein, we used a Mendelian randomization (MR)-based approach to identify skipped exons (the predominant splicing event in brain) that contribute to AUD risk. Genotypes and RNA-seq data from the CommonMind Consortium were used as the training dataset to develop predictive models linking individual genotypes to exon skipping in the prefrontal cortex. We applied these models to data from the Collaborative Studies on Genetics of Alcoholism to examine the association between the imputed *cis*-regulated splicing outcome and the AUD-related traits. We identified 27 exon skipping events that were predicted to affect AUD risk; six of these were replicated in the Australian Twin-family Study of Alcohol Use Disorder. Their host genes are *DRC1*, *ELOVL7*, *LINC00665*, *NSUN4*, *SRRM2* and *TBC1D5*. The genes downstream of these splicing events are enriched in neuroimmune pathways. The MR-inferred impacts of the *ELOVL7* skipped exon on AUD risk was further supported in four additional large-scale genome-wide association studies. Additionally, this exon contributed to changes of gray matter volumes in multiple brain regions, including the visual cortex known to be involved in AUD. In conclusion, this study provides strong evidence that RNA alternative splicing impacts the susceptibility to AUD and adds new information on AUD-relevant genes and pathways. Our framework is also applicable to other types of splicing events and to other complex genetic disorders.

## Introduction

RNA alternative splicing is known to be associated with many complex diseases, especially neurological or brain disorders, including alcohol use disorder (AUD) [1–3]. AUD is a prevalent psychiatric disorder characterized by problems resulting from excessive and compulsive alcohol consumption. In the United States, AUD affects nearly 29.5 million individuals and is the third-leading preventable cause of death [4, 5]. There is a genetic component to the risk for AUD, with the estimated heritability ranging from 40% to 60% [6, 7]. Genome-wide association studies (GWAS) have identified many AUD-associated genetic variants and genes, including genes encoding the alcohol metabolizing enzymes ADH1B and ADH1C, zinc transporter SLC39A8, and neurotransmitter receptor DRD2 [8–11]. Genome-wide changes in RNA splicing were recently reported in multiple human brain regions in individuals with AUD [12, 13]. While previous research has identified alternatively spliced mRNAs induced by alcohol, whether alternative splicing impacts the susceptibility for AUD is not well studied.

The major challenge of studying RNA splicing in AUD is the scarcity of large-scale transcriptomic data with high sequencing depths in brains from individuals with and without AUD. Moreover, the contribution of an RNA splicing event to risk for AUD cannot be directly inferred using RNA sequencing (RNA-seq) alone [14], because the splicing changes contributing to the disorder cannot be distinguished from the splicing changes induced by alcohol exposure. Currently, methods are available to infer the causality of gene expression for a trait, such as PrediXcan [15], transcriptome-wide association study (TWAS) [16], and summary-based Mendelian randomization (SMR) [17]. These methods have been used in combination with splicing quantitative trait loci (sQTL) to study the causal effect of alternative splicing in the susceptibility to diseases such as Alzheimer’s disease [18], glioma [19], osteoporosis [20], and more recently, AUD [13]. Although these studies suggest that alternative splicing of genes is associated with complex diseases including AUD, identification of specific splicing events would provide not only stronger evidence that RNA alternative splicing impacts risk for AUD, but also the molecular basis for experimentally verifying the causal nature of RNA alternative splicing. To identify these splicing events, it is necessary to quantify the proportion of splicing outcome that is genetically determined and evaluate the contribution of this proportion to AUD risk.

In this study, our primary aim was to identify alternatively spliced exons that contribute to the susceptibility to AUD. To achieve this aim, we established a computational model to predict *cis*-regulated exon skipping in dorsolateral prefrontal cortex (DLPFC), using data from the CommonMind Consortium (CMC) [21]. Using the resulting prediction models, potentially causal skipped exons were identified by a Mendelian randomization (MR)-based approach that examined the association between the genotype-imputed *cis*-regulated components of exon skipping and DSM-IV alcohol dependence and symptom counts, based on individual genotype and phenotype data from the Collaborative Study on the Genetics of Alcoholism (COGA) [22–24]. Significant results were evaluated in a replication dataset of the Australian Twin-family Study of Alcohol Use Disorder (OZALC) [25]. In particular, an alternatively spliced non-coding exon in *ELOVL7* was found to impact AUD susceptibility; its impact was further evaluated by leveraging the power of additional large-scale GWAS. Furthermore, the impact of the *ELOVL7* skipped exon in the brain was visualized by association analysis with magnetic resonance imaging data from the UK Biobank. Predicted downstream genes and biological pathways of the replicated splicing events implicated immunological and neurological functions. The framework presented here is broadly applicable to study the role of RNA splicing in the heritability of complex disease.

## Materials and methods

### Dataset for splicing model development

RNA sequencing data from the dorsolateral prefrontal cortex (DLPFC) and DNA genotyping data from 991 samples were downloaded from the CommonMind Consortium (CMC) [21]. The RNA-seq data were processed as described previously [26]. Genetic variants that had minor allele frequency (MAF) ≥ 0.03, Hardy–Weinberg equilibrium *P* > 0.001, and genotyping rate ≥0.95, were used as the input for imputation using the Michigan Imputation Server with default parameters. The reference panel used was 1000 G Phase 3 v5 (GRCh37.p13, hg19; EUR). EAGLE was used to phase genotypes and Minimac4 (v1.2.1) was used for imputation [27].

### Quantification of exon inclusion

The outcome of alternative splicing was quantified as percent-spliced-in (PSI, Ψ) computed by *replicate Multivariate Analysis of Transcript Splicing* (rMATS, version 4.0.2) [28]. We adopted the Gencode annotation (GTF, GRCh38.p13, hg38) to determine the exon skipping events. GTF annotations were converted from hg38 to hg19 using *LiftOver* (version 1.20.0) [29] to be consistent with the genotype data. Using the RNA-seq alignment files, junction reads supporting the inclusion or exclusion isoforms of all the annotated exon skipping events were counted. PSI was calculated as,

$$\Psi = \left( {\frac{I}{{L_I}}} \right)\bigg/\left( {\frac{I}{{L_I}} + \frac{S}{{L_S}}} \right)$$

where *I* and *S* are the junction read counts supporting the inclusion and exclusion (skipped exon) isoforms, respectively. *L*_*I*_ and *L*_*S*_ are the effective lengths of the exon inclusion and exclusion isoforms, respectively, which were automatically calculated by rMATS based on the annotation GTF.

### Modeling PSI using genotype

To quantify the component of PSI (Ψ) determined by *cis*-acting genetic variants, we established a computational model based on the genotype and PSI derived from RNA-seq data of 991 CMC subjects [21]. For modeling, we required that each splicing event must have: (i) more than one SNV located in the transcribed region of the gene; (ii) number of support samples ≥100; and (iii) interquartile range (IQR) of the calculated Ψ across all support samples >10%. Specifically, support samples were defined as those having genotype information available and total junction read counts (including both inclusion and exclusion events) ≥10. For each splicing event, we retrieved all SNVs within the transcribed region spanning from the transcription start site (TSS) to the end of 3’-untranslated region (UTR) of the host gene with MAF ≥ 0.01 and genotype imputation score ≥0.6. To maximize the probability that the SNVs included in each model were informative and to reduce the computation complexity for events with large numbers of SNVs in the transcribed region, we selected up to 20 top SNVs based on the ranking of their genotype correlation with Ψ. Next, we used the elastic net regularization algorithm to determine which variants were predictive for each splicing event.

The variants selected by elastic net were used to calculate the genetically determined component of Ψ, i.e., $\hat \Psi$,

$${{{\hat{\mathrm \Psi }}}} = \alpha + \mathop {\sum }\limits_{k = 1}^N \beta _kX_k + \varepsilon$$

where *α* is the intercept (basal level), *β*_*k*_ is the coefficient (weight or effect size) of SNV *k* with alternative allele dosage *X*_k_, *ε* is the noise, and *N* ≤ 20.

For each splicing event, the model performance was further evaluated by leave-one-out cross-validation, in which the model was established using n-1 samples and the splicing outcome of the n^th^ sample was predicted. We calculated the Pearson’s correlation ***r*** between the cross-validated ${{{\hat{\mathrm \Psi }}}}$ and the observed Ψ. The *p*-value of the correlation was used as an indicator of how reliably the genetic variants explained the Ψ to the extent measured by ***r***. Importantly, since ***r*** is the correlation between prediction and observation, a zero or negative value indicates that the model is non-explanatory. Thus, to test whether the Ψ of each splicing event is explainable by genetic variants, the *p*-value was calculated as $P\left( {H_0:r \le 0, \cdot H_a:r \, > \, 0} \right)$. Any splicing event with a greater significance than the Bonferroni adjusted 5%-threshold was accepted for later analysis. In addition, the coefficient of determination, ***R***^**2**^, was calculated as:

$$R^2 = 1 - {\sum} {\left( {{{{\hat{\mathrm \Psi }}}}_i - {{\Psi }}_i} \right)^2/\left( {{{{\bar{\mathrm \Psi }}}} - {{\Psi }}_i} \right)^2}$$

where ${{{\hat{\mathrm \Psi }}}}_i$ and Ψ_*i*_ are the predicted and observed splicing outcomes of individual *i*, respectively. ${{{\bar{\mathrm \Psi }}}}$ is the average across all individuals.

### Heritability estimation for PSI

For each splicing event, Genome-wide Complex Trait Analysis (GCTA) [30] was used to estimate heritability (***h***^**2**^) of the PSI (Ψ), i.e., proportion of variance in Ψ explained by all the genetic variants genome-wide. The GCTA-GREML approach was performed with Ψ as the molecular trait and using all genetic variants in the CMC samples having a genotype imputation score ≥0.6 and MAF ≥ 0.01. The estimation of ***h***^**2**^ was adjusted for the covariates sex and sequencing cohort.

### Independent RNA-seq data used for validating elastic net-derived splicing models

We used genotype and RNA-seq data from 139 human postmortem brain samples of the superior frontal gyrus (Brodmann area 8) that were previously reported [31]. PSI of the modeled events were quantified using rMATS with Gencode annotation (GRCh37.p13, hg19). To compare the analysis from this cohort with CMC, the same standards were imposed. Skipped exons that have: (i) all marker variants available in the genotypes of the PFC samples; (ii) more than 100 samples with ≥10 junction reads; and (iii) PSI with IQR > 0.1, were compared.

### GWAS data from the Collaborative Studies on Genetics of Alcoholism (COGA)

COGA is a family study that includes both genotypic and alcohol-related phenotypic data [22, 23]. Genotyping and imputation were previously described [24]. We selected variants identified as PSI-predictive in the CMC elastic net models. We focused on 8,038 European American (EA) individuals from 1127 independent families, the largest ancestry group in COGA. The phenotypes used in this analysis were DSM-IV alcohol dependence (1 if dependent and 0 if non-dependent) and symptom count (SXCT, the number of DSM-IV criteria met by a participant; range from 0 to 7) [24]. We adjusted for 11 covariates: sex, 3 genotyping array platforms, 4 principal components of population stratification, and 3 birth cohorts [24]. Because the COGA genotyping arrays differed from CMC, the imputation might result in different variants. When imputing the PSI of the skipped exon from the COGA genotype data, we only used the variants that were present in both datasets, which was over 90% of the original CMC variants.

### Identification of exon skipping events contributing to the susceptibility for AUD

A Mendelian randomization (MR)-based approach was designed to examine the relationship between the PSI imputed from genotypes and the GWAS AUD trait. In our implementation, the genetic variant (*x*) was the instrumental variable encoding the information from the DNA level. The genotype-imputed PSI ${{{\hat{\mathrm \Psi }}}}_{\left( x \right)}$ was an intermediate molecular trait (equivalent to the exposure in classic MR literature) [32]. Finally, the AUD phenotype was the outcome (*y*). A significant association between ${{{\hat{\mathrm \Psi }}}}_{\left( x \right)}$ and *y* indicates that genetic variants contribute to the outcome (AUD) via RNA splicing.

Using the COGA data, we examined the association between the imputed PSI ${{{\hat{\mathrm \Psi }}}}_{\left( x \right)}$ and both DSM-IV alcohol dependence and SXCT using *generalized estimating equation* (GEE) [24, 33]. Binomial (logit link function) and Poisson (log link function) were assumed to model DSM-IV AUD and SXCT, respectively. The pedigree matrix was constructed as a tiling of blocks along the diagonal; each block contained the correlation coefficients of individuals from an independent family. Equal coefficients were assumed for individuals in the same family; coefficients between individuals from different families were zero. Finally, the GEE regression was further adjusted with covariates of sex, 3 genotype arrays, 4 principal components related to population, and 3 birth cohorts.

### Replication using data from the Australian Twin-family Study of Alcohol Use Disorder (OZ-ALC)

This dataset, including genotypes and DSM-IV alcohol-dependence phenotypes, was downloaded from dbGaP (phs000181.v1.p1) [25]. As in COGA, we limited the replication analysis to European American (EA) individuals (*n* = 2856), and the pedigree matrix was constructed in the same way. Additionally, sex, age, and the first three principal components of population stratification as specified by OZ-ALC were included as covariates in the replication analysis.

### Analysis of downstream differentially expressed genes

We stratified the CMC samples according to the high and low levels of the genetically determined ${{{\hat{\mathrm \Psi }}}}$, for each skipped exon identified in COGA and replicated in OZ-ALC. Read counts for the respective groups of samples (G1: low ${{{\hat{\mathrm \Psi }}}}$; G2: high ${{{\hat{\mathrm \Psi }}}}$) were retrieved from the RNA-seq data and a gene-by-sample read count matrix was constructed. We considered only the autosomal genes and removed low expression genes, which were defined by ≤ 1 CPM in more than *N* samples, where $N = \frac{1}{2}min\left( {n_1,n_2} \right)$; *n*_*1*_ and *n*_*2*_ are the sample numbers in G1 and G2, respectively. We used the TMM method in the R package EdgeR (version 3.34.1) [34] to normalize the read counts. Differentially expressed genes were identified in EdgeR using a negative binomial model with adjustments for two covariates: sex and sequencing cohort. Cutoff of significance was FDR < 0.05.

### Pathway enrichment analysis

We used the R package ClusterProfiler (version 4.0.5) [35] to perform enrichment analysis for the differentially expressed genes based on Gene Ontology (GO) biological processes and molecular functions. The enrichment significance threshold was FDR < 0.05. We further explored the functions of the skipped exons by Gene Set Enrichment Analysis (GSEA) [36] through ClusterProfiler using three pathway knowledgebases GO [37], KEGG [38] and Hallmarks [39]. An enrichment score was computed for each pathway to determine if it was enriched or depleted based on changes of the ${{{\hat{\mathrm \Psi }}}}$ levels. The significance threshold was also FDR < 0.05.

### Additional GWAS datasets

Summary statistics from four large-scale GWAS were downloaded: (i) Psychiatric Genomics Consortium analysis of DSM-IV alcohol dependence [9] (PGC, *n* = 52,848, EU and AA ancestries); (ii) GWAS and Sequencing Consortium of Alcohol and Nicotine Use (GSCAN, *n* = 941,280, EU ancestry) analysis of drinks per week (DrnkWk) [40]; (iii) Million Veteran Program (MVP, *n* = 274,424, multiple ancestries including EU and AA) analysis of AUD diagnosis based on International Classification of Diseases 10th Revision (ICD-10) [41]; and *iv*) UK Biobank (UKB, *n* = 112,117, EU ancestry) analysis of AUDIT-P (Problems) scores [42].

### GWAS statistics summary-based analysis

For the gene *ELOVL7*, we extracted all genetic variants (*x*) located in the transcribed region (from TSS to 3’UTR) from the CMC genotyped subjects. The effect size *β*_*xy*_ of each variant (*x*) on the AUD trait (*y*) was retrieved from the GWAS summary statistics. The effect size *β*_*xΨ*_ of each variant (*x*) on PSI (Ψ) of the *ELOVL7* exon skipping event was calculated based on CMC data, using a linear regression model adjusted for the demographic covariates of sex and ethnic group. To infer the causality of splicing (Ψ) on trait (*y*), we co-localized *β*_*xy*_ with *β*_*xΨ*_ by Generalized Summary data-based Mendelian Randomization (GSMR), in which the causal effect size of Ψ on *y*, i.e., $\hat \beta _{{{\Psi }}y}$ was estimated by a least-square (LS) regression model [43].

### Brain magnetic resonance imaging (MRI) analysis

The T1 structural MRI data of subcortical volumes (FIRST, *n* = 14), regional gray matter volumes (FAST, *n* = 139), and genotypes for 21,402 subjects were downloaded from UKB [44]. The ${{{\hat{\mathrm \Psi }}}}$ of the *ELOVL7* skipped exon was imputed for each subject and the ${{{\hat{\mathrm \Psi }}}}$ was regressed against each of the FIRST and FAST volumes. The regression was conducted using generalized linear model (GLM) with Gaussian (log link function) and adjusted for three covariates: sex, age and education score specified by UKB [45]. FDR values of significant changes in the volumes were mapped to the Desikan-Killiany atlas [46] to visualize the regions of interest.

## Results

### Predictive models for the genetic components of alternative splicing

**Fig. 1:**
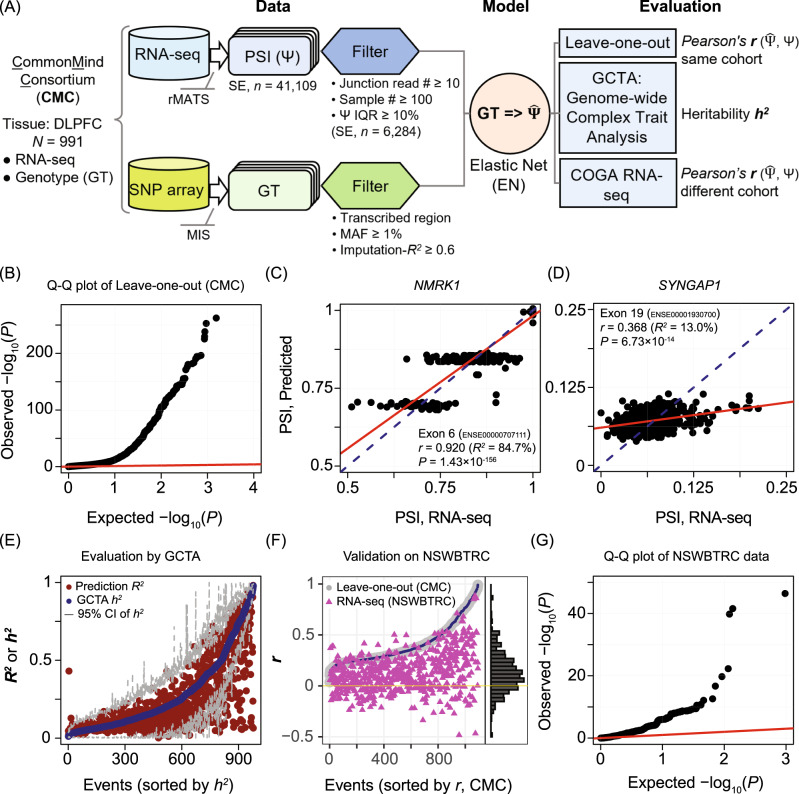
Predictive modeling for the genetic component of skipped exon (SE) events. **A** Modeling workflow. CommonMind Consortium (CMC) RNA-seq and genotyping data from dorsolateral prefrontal cortex (DLPFC) were used to derive the splicing outcomes (PSI, Ψ) and imputed genotypes (GT), respectively. These data were filtered before training the elastic net (EN) model that was used to compute the genetically determined component of Ψ, denoted as ${{{\hat{\mathrm \Psi }}}}$. The models were evaluated by leave-one-out cross validation, Genome-wide Complex Trait Analysis (GCTA) and a replication RNA-seq dataset from the New South Wales Brain Tissue Resource Center (NSWBTRC). MIS, Michigan imputation Server. **B** Quantile-quantile (Q-Q) plot of leave-one-out. Observed significance (-log_10_ *P* value, black dots, *n* = 6284 SE) of the Pearson’s ${{{{{{{\boldsymbol{r}}}}}}}}\left( {{{{\hat{\mathrm \Psi }}}},{{\Psi }}} \right)$ against a random null distribution (red line) in CMC. **C** Example of a highly *cis*-regulated splicing event. The genetically predicted PSI (y-axis) is plotted versus the total PSI derived from RNA-seq (x-axis) for a specific SE (*ENSE00000707111*) in *NMRK1* using the CMC samples (black dots, *n* = 380). Pearson’s *r* and its *P* value; and the *R*^2^, proportion of PSI variance explained by the model are provided. Solid red line represents the correlation. Dashed blue line is the identity line. **D** Example of a lowly *cis*-regulated splicing event. The genetically predicted PSI (y-axis) is plotted versus the total PSI derived from RNA-seq (x-axis) for a specific SE (*ENSE00001930700*) in *SYNGAP1* using the CMC samples (black dots, *n* = 380). Pearson’s *r* and its *P* value, and the *R*^2^ are provided. Solid red line represents the correlation. Dashed blue line is the identity line. **E** Model evaluation by heritability analysis. The finalized elastic net models (1093 SE) were evaluated by the independent heritability analysis approach of GCTA. Results shown are the model prediction (*R*^2^, red dots), GCTA evaluation (*h*^2^, blue dots in ascending order), and 95% confidence interval (CI) of *h*^2^ (gray dashes), for each event. For 87.5% of the splicing events, our predicted *R*^2^ lies between the lower and upper bounds of the GCTA estimation *h*^2^, indicating that the model prediction is consistent with genome-wide estimation. **F** Model validation on replication cohort. The same elastic net models as in (E) were validated on the NSWBTRC RNA-seq cohort, which contains different individuals from CMC. Leave-one-out Pearson’s *r* from our models are shown (gray dots, ascending order), in which the eligible events for comparison in the replication cohort are highlighted in blue (570 SE). The replication Pearson’s *r* are shown as purple triangles. Distribution of the replication *r* is visualized by the marginal histogram, where 75.4% of the events had a *r* > 0 (yellow horizontal line) indicating the success of replication. **G** Quantile-quantile (Q-Q) plot based on the replication cohort. Observed significance (-log_10_ *P* value, black dots, *n* = 570 SE) of the Pearson’s *r* against a random null distribution (red line) in the COGA RNA-seq cohort.

In this study, we focused on skipped exons (SE), the dominant type of splicing event in the brain, including in the prefrontal cortex [47, 48]. A predictive model was built for each SE to determine the extent that genetic variants could explain the splicing outcome. We predicted the genetically determined inclusion levels (${{{\hat{\mathrm \Psi }}}}$) for a total of 41,109 SE events annotated in Gencode using the RNA-seq data and imputed genotypes from the CommonMind Consortium (CMC) [21]. The overall workflow is depicted in Fig. 1A. After filtering for the number of junction reads (>10), number of samples (>100), and PSI variability (IQR > 10%), there were 6284 SE events remaining for analysis. For each SE, we used a semi-supervised method to select the SNVs that were most explanatory of the PSI variability. Then we applied the elastic net algorithm to determine the marker SNVs for PSI prediction. Although we initiated the modeling using more relaxed criteria, we found that all of the final selected variants had MAF ≥ 5%, and 90% of them had imputation scores over 0.8, indicating that our approach converged on higher confidence SNVs.

To evaluate how much the genetic variants could explain the splicing outcome for each SE, we performed leave-one-out cross validation. The SNV-determined proportion in the PSI of an exon skipping event was assessed using Pearson’s correlation ***r*** between the predicted PSI (${{{\hat{\mathrm \Psi }}}}$) and RNA-seq measured PSI (Ψ). An SE with a significant *p* value for a positive ***r*** indicates that the splicing outcome can be, at least partially, explained by the SNVs in the transcribed region. The variability of the PSI explained by the model, *R*^2^, was also calculated (Supplementary Fig. S1). Figure 1B shows the quantile-quantile plot of the observed *p* values from our models (cross validated) against the expected *p* values under the null hypothesis, which were randomly drawn from a uniform distribution ranging from 0 to 1. We observed a substantial deviation from the null distribution, indicating that exon inclusion of a large proportion of skipped exon events in the DLPFC transcriptome can be partially explained by the genetic variants. We used a Bonferroni *p* cutoff = (1/6284) × 0.05 = 7.96 × 10^−6^ for the significance of *cis*-regulation because our subsequent Mendelian randomization requires strong dependency of splicing outcome on genetic variants. This resulted in 1093 SE events.

We found that the degree to which exon skipping was genetically determined varied widely. For example, in *NMRK1* (*Nicotinamide Riboside Kinase 1*), a key enzyme in the synthesis of NAD+, the PSI of exon *ENSE00000707111* was highly *cis*-regulated (*R*^2^ = 0.847, Fig. 1C). In contrast, in *SYNGAP1* (*Synaptic Ras GTPase Activating Protein 1*), a gene associated with AUD and involved in regulating synaptic plasticity and neuronal homeostasis [11], exon *ENSE00001930700* showed a low degree of *cis*-regulation (*R*^2^ = 13.5%, Fig. 1D). Our results are consistent with the notion that complex regulatory mechanisms influence splicing outcomes and that genetic variants are only one of several contributing factors.

### Validation of predictive models

To test whether the marker variants from the elastic net models were appropriately determined, we evaluated the model predictions for the 1,093 events by estimating the heritability of the splicing outcomes using GCTA [30]. We found that the ***R***^**2**^ of the model prediction for 87.5% of the events were within the 95% confidence interval (CI) of the GCTA estimated heritability, ***h***^**2**^ (Fig. 1E), which indicates that the genetically determined splicing outcomes can be largely explained by the variants we selected within the transcribed region.

We carried out a replication analysis by predicting the PSI in an independent, previously reported RNA-seq dataset from superior frontal gyrus of subjects from the New South Wales Brain Tissue Resource Center (NSWBTRC) [31]. PSI of each SE was predicted based on the genotypes of the NSWBTRC subjects using the models established from CMC. Using the same criteria as for the CMC cohort resulted in 570 SE, which were used to compare the model predictions from the CMC and the NSWBTRC cohorts. We found that the predictions from the NSWBTRC dataset was consistent with that of CMC (Fig. 1F). The majority (75.4%) of the results showed positive correlation between the genetically imputed and the RNA-seq PSI. Similar to the CMC result (Fig. 1B), the *p* value distribution deviated from the null hypothesis of randomness (Fig. 1G).

### Identification of SE contributing to AUD susceptibility

**Fig. 2:**
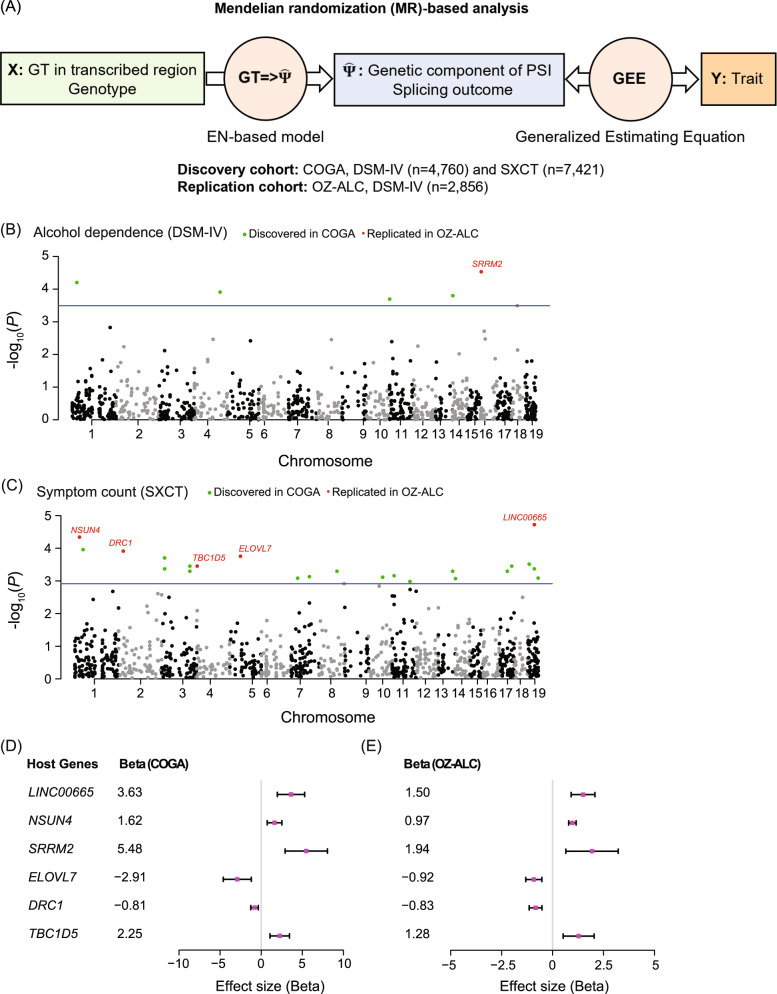
Mendelian randomization (MR)-based analysis of COGA. **A** Overview of the MR-based analysis. Genetic variant (*X*) is the instrumental variable. The intermediate molecular trait (i.e., exposure) is the genetically predicted PSI, ${{{\hat{\mathrm \Psi }}}}_{\left( x \right)}$, for RNA splicing, and the phenotypic variable is the trait (*Y*). ${{{\hat{\mathrm \Psi }}}}$ is inferred from *X* using the elastic net (EN) models and the association between ${{{\hat{\mathrm \Psi }}}}$ and *Y* is evaluated by generalized estimating equation (GEE). Splicing events showing significant associations with the trait *Y* are putatively causal for the trait. This MR pipeline was run in the discovery cohort COGA and repeated in the replication cohort OZ-ALC. The number of subjects for each phenotype is provided. **B**, **C** Manhattan plots of significant splicing events. Chromosomal distribution of significance of association for all splicing events (${{{\hat{\mathrm \Psi }}}}$, *Y*), with respect to the DSM-IV (**B**) and SXCT (**C**). Blue line, -log_10_ of the *p*-value corresponding to FDR = 0.05; green dots, significant events in discovery cohort; red dots, replicated significant events. **D**, **E** Effect sizes of the replicated events. Forest plots of the effect sizes of the six replicated events. The estimates of effect sizes (beta) in COGA (**D**) and OZ-ALC (**E**) are consistent. The rectangles are the estimates and hashed lines represent the 95% CI.

To test whether these skipped exon events play a causal role in the development of AUD, we designed a MR-based approach. First, considering the genetic variants as the instrumental variable, we imputed the splicing outcome ${{{\hat{\mathrm \Psi }}}}$ for each of the 1093 SE using our predictive models, based on the genotypes of 8038 EA subjects from 1127 independent families in COGA [24]. Second, we examined the associations between ${{{\hat{\mathrm \Psi }}}}$ and alcohol dependence diagnosis (DSM-IV, *n* = 2348 control and 2412 AUD subjects) and symptom count (SXCT, *n* = 7421; 67% had one or more symptoms). The analysis workflow is depicted in Fig. 2A.

Of the 1093 SE, we found that five were significantly associated with DSM-IV dependence (Fig. 2B) and 24 were significantly associated with SXCT (Fig. 2C); two events were associated with both DSM-IV and SXCT. Overall, the result of our MR-based analysis indicated that 27 SE events contributed to at least one of these AUD traits (Supplementary Table S1).

We next asked whether these 27 events could be replicated in an independent dataset. To address this question, we conducted the same analysis using the Australian Twin-family Study of Alcohol Use Disorder (OZ-ALC) dataset, which included 2856 individuals [24]. We found that six of the 27 SE were replicated with FDR < 0.05; these events were among the top candidates ranked by *p*-value from the COGA discovery cohort (Fig. 2B, C). Moreover, the effect sizes of all six SE were consistent in both the COGA and OZ-ALC cohorts (Fig. 2D, E). Detailed information for the 6 events is summarized in Table 1.

**Table 1:** Causal exons for alcohol use disorder.

Skipped exon (SE)				Exon function			Predictive modeling				Discovery		Replication
SE	Host gene	Exon	Chromosome	Effects host gene expression	Protein coding	Functional domain (Pfam)	Num. of samples (CMC)	Num. of variants	*P*-value	R^2^	Phenotype	FDR	FDR
1	*LINC00665*	ENSE00002438745	chr19	No	No	N.A.	380	3	3.42E-87	64.4%	SXCT	0.021	0.005
2	*NSUN4*	ENSE00001875548	chr1	Yes	No	N.A.	737	2	3.75E-42	22.1%	SXCT	0.031	0.005
3	*SRRM2*	ENSE00002674786	chr16	No	No^a^	No	380	6	4.86E-06	3.1%	DSM-IV	0.032	0.029
4	*ELOVL7*	ENSE00002079807	chr5	Yes	No	N.A.	380	11	9.13E-42	39.5%	SXCT	0.031	0.033
5	*DRC1*	ENSE00003572542	chr2	Yes	Yes	PF14772	338	15	5.99E-39	38.9%	SXCT	0.031	0.033
6	*TBC1D5*	ENSE00001693995	chr3	N.A.	No	N.A.	376	14	1.66E-27	26.4%	SXCT	0.031	0.033

Genome assembly: GRCh38/hg38. PF14772: Dynein regulatory complex protein 1/2, N-terminal domain. DSM-IV indicates alcohol dependence diagnosis. SXCT: symptom count.

Sample numbers for SXCT and DSM-IV in discovery cohort are 7421 and 4760, respectively. Replication sample number is 2856.

^a^Contains a stop codon triggering nonsense mediated decay.

The host genes for these six SE include one lncRNA (*LINC00665*) and five protein-coding genes: *NSUN4* (NOP2/Sun RNA Methyltransferase 4), *SRRM2* (Serine/Arginine Repetitive Matrix 2), *ELOVL7* (Elongation of Very Long Chain Fatty Acids Protein 7), *DRC1* (Dynein Regulatory Complex Subunit 1) and *TBC1D5* (TBC1 Domain Family Member 5). We searched the GTEx database and found that all six genes are expressed in the brain. We also found evidence in the literature that each of the six genes plays roles in alcohol-related diseases, neurological disorders, or immune response [49–55]. In addition, the elastic net models specified 51 genetic variants that are most explanatory to the PSI of the six SE (Supplementary Table S2). None of these explanatory variants have previously been associated with AUD in the NHGRI GWAS Catalog [56] or in a recent comprehensive genome-wide meta-analysis of problematic alcohol use [11].

### Predicted downstream genes regulated by the identified SE

To further explore the function of the six identified SE in human brains, we designed a computational strategy to identify their downstream genes. For each SE, we first predicted the ${{{\hat{\mathrm \Psi }}}}_{SE}$ using the genotypes in the transcribed region of the host gene from all available CMC samples, and then we stratified the individuals into two groups based on low and high ${{{\hat{\mathrm \Psi }}}}_{SE}$.levels (Supplementary Fig. S2A–E). The splicing event in*TBC1D5* was not analyzed because most samples clustered in the central region of all ${{{\hat{\mathrm \Psi }}}}$ values; thus, the number of remaining samples that could be stratified as high or low was insufficient for statistical analysis (Supplementary Fig. S2F). We identified the differentially expressed genes between the two groups for each SE independently. The number of the downstream differentially expressed genes identified for each SE ranged from 5 for *LINC00665* to 471 for *DRC1* (Supplementary Fig. S3). In total, 970 unique differentially expressed genes were found; the full list of differentially expressed genes for each SE is provided in Supplementary Table S3.

We next used these 970 differentially expressed genes to provide additional information regarding causality to 4456 genes previously found to be responsive to alcohol in a cell culture study [57]. We found 197 genes were also differentially expressed following alcohol treatment in a lymphoblastoid cell line. Of these genes, 173 (88%) are expressed in human brain. In particular, two genes (*OXTR* and *OAS3*) showed evidence for association with alcohol dependence or consumption in GWAS (at *p* ≤ 9 × 10^−6^) [58, 59]; 48 genes were differentially expressed in at least one human brain region between alcohol dependence and control individuals; and 55 genes were differentially expressed in the brains of selectively bred alcohol-preferring (P) rats consuming large amounts of alcohol [57, 60]. Twenty genes overlapped between the 48 differentially expressed genes in human post-mortem brain and 55 differentially expressed genes in P rat brain studies (Supplementary Table S4). Therefore, these 20 genes might be prioritized in future experimental studies.

**Fig. 3:**
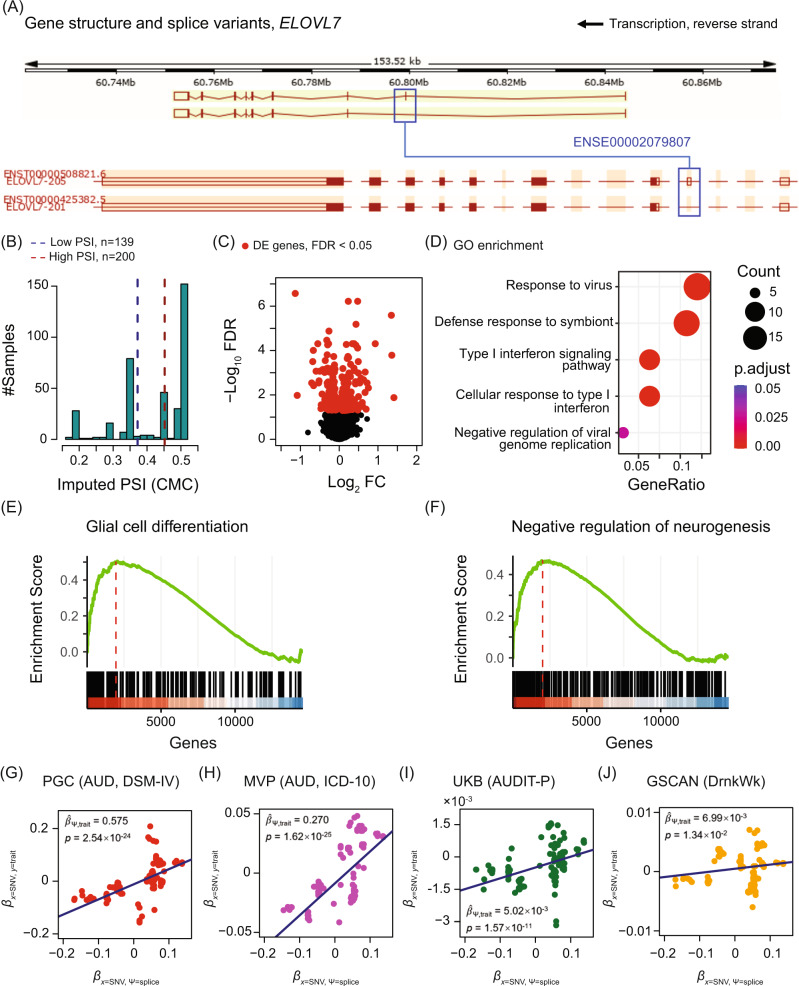
Functional analysis exemplified by the *ELOVL7* splicing event. **A** Schematic of two *ELOVL7* splice variants. Splicing pattern and gene structure were adapted from Ensembl genome browser. The skipped exon (SE) is highlighted. Open and filled boxes represent untranslated and protein coding regions, respectively. **B** Sample stratification. CMC samples with genetically imputed PSI values greater than the level marked by the red dashed line (*n* = 200) were labeled as high, and those less than the level marked by the blue dashed line (*n* = 139) were labeled as low. Intervening samples (*n* = 41) were unused. **C** Differentially expressed (DE) genes. Volcano plot shows the -log_10_ FDR (y-axis) versus the log_2_ fold-change (FC, x-axis). Red dots are the differentially expressed genes between the high and low PSI groups in (**B**) with FDR < 0.05. **D** Gene Ontology (GO) pathway enrichment of DE genes. Pathways enriched by the DE genes with FDR < 0.05 are shown with the respective gene ratios. The color represents the FDR, i.e., the Benjamini-Hochberg-adjusted *p* values. The size of dots indicates the gene count. **E**, **F** Examples of two neural pathways enriched in GSEA. These pathways were enriched by genes upregulated in samples having high PSI level. The green line is the running enrichment score and the red dished line marks the maximum of score that corresponds to the leading-edge subset of genes that optimally contribute to the enrichment. Genes (black bars) were ranked high (red) to low (blue) based on log_2_ FC between the high and low PSI groups in (**B**). The normalized enrichment score (NES) and the FDR of enrichment are shown. **G**–**J** Results of Generalized Summary data-based Mendelian Randomization (GSMR). Effect sizes of SNV (x) on trait (y), *β*_x,y_, were plotted versus the effect sizes of SNV (x) on splicing (Ψ), *β*_*x*,Ψ_. The estimated slope ($\hat \beta _{{{\Psi }},y}$), which is the coefficient of least-square (LS) regression, is shown with the *p* value. **G** Psychiatric Genomics Consortium (PGC) GWAS of alcohol dependence (AD, DSM-IV). **H** Million Veteran Program (MVP), alcohol use disorder (AUD, ICD10). **I** UK Biobank (UKB), problematic drinking (AUDIT-P). **J** GWAS and Sequencing Consortium of Alcohol and Nicotine Use (GSCAN), drinks per week (DrnkWk).

The host gene for one of the identified SE events, *ELOVL7*, was among the 20 genes common to both the human and rat studies. This event had the largest variance for the genetically imputed PSI (${{{\hat{\mathrm \Psi }}}}$), thereby incurring high statistical power for MR causality inference [32, 61]. The *ELOVL7* skipped exon (ENSE00002079807) is in the 5’UTR (Fig. 3A) and it does not change the protein sequence. We found that the change of ${{{\hat{\mathrm \Psi }}}}$ for this SE (Fig. 3B) correlated with differential expression of *ELOVL7* itself, and 249 other genes (FDR < 0.05, Fig. 3C and Supplementary Table S3). Another notable example for AUD relevance is the gene *heat shock protein family A (Hsp70) member 6* (*HSPA6*), which encodes a splicing factor found to be significantly upregulated in human brain upon alcohol intake [12]. *HSPA6* was identified as a downstream gene of the SE in *ELOVL7* as well as the events in *LINC00665* and *NSUN4*.

### Pathway analyses of the differentially expressed genes downstream of the six skipped exons

GO enrichment analysis (Supplementary Table S5) showed that the *ELOVL7* splicing event implicated 250 differentially expressed genes that significantly enriched immune response pathways (Fig. 3D), such as, the type I interferon (IFN) signaling pathway (FDR = 7.20 × 10^−06^). Gene Set Enrichment Analysis (GSEA) [39] of three pathway databases (GO, KEGG, and MSigDB Hallmark) showed consistent enrichment for these immune pathways (Supplementary Table S6) along with several neural pathways, including glial cell differentiation and neurogenesis regulation (Fig. 3E, F). Neuroimmune pathways were commonly enriched for multiple SE events, including TNF, NF- κB, IL6-JAK-STAT3, IL2-STAT5, NOD-like receptor (NLR) signaling pathways, as well as T cell activation and differentiation (Supplementary Table S6). Noteworthy, the complement cascade, which is part of the innate immune system involved in alcoholic liver disease [62], was enriched for the SE events in *ELOVL7, LINC00665*, *NSUN4*, and *SRRM2*. We also found that epithelial-mesenchymal transition (EMT) was enriched for the SE events in *ELOVL7*, *SRRM2* and *TBC1D5* and was depleted for *DRC1 and LINC00665* in GSEA. Recent studies suggest that genes associated with EMT have altered expression levels in the brain of patients with Alzheimer’s disease, which causes chronic neuroinflammation [63]. Other functions significantly enriched or depleted for the identified events included protein folding, chaperone (modulatory process), heat response and heat shock protein, cell-cell adhesion, ECM-receptor signaling pathways, and autoimmune disease (e.g., diabetes).

### GWAS summary data-based analysis

To test the reproducibility of the genetically inheritable effect of the *ELOVL7* SE event on AUD, we performed additional analyses in four large-scale AUD-related GWAS datasets (PGC, MVP, UKB, and GSCAN), using Generalized Summary data-based Mendelian Randomization (GSMR) [43]. Using CMC as the training set, GSMR inferred the causality of the *ELOVL7* SE event on four AUD-related traits: DSM-IV alcohol dependence, ICD-10 AUD diagnosis, AUDIT-P, and drinks per week (Fig. 3G–J). These results showed significant causality in each GWAS dataset, indicating that the splicing regulation of *ELOVL7* likely plays an important role in the genetic basis of AUD.

### Association analysis with brain MRI data

**Fig. 4:**
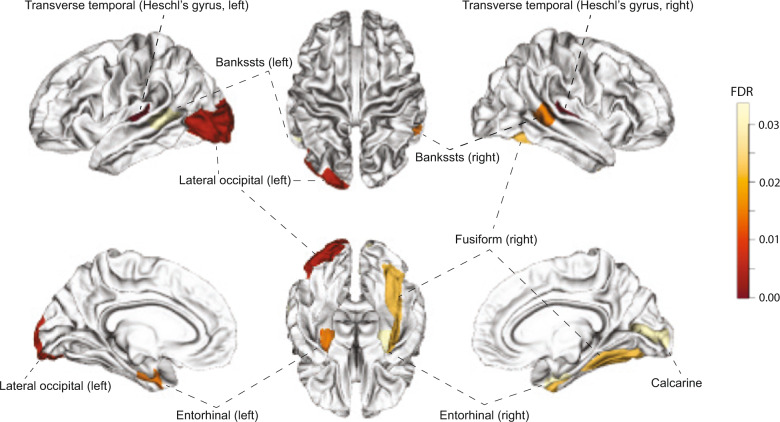
Impact of the *ELOVL7* exon skipping event in the brain. Regions showing significant changes of gray matter volume in UK Biobank (UKB) subjects with high *cis*-regulated PSI (${{{\hat{\mathrm \Psi }}}}$) compared with individuals having low ${{{\hat{\mathrm \Psi }}}}$. FDR of the changes were mapped to the Desikan–Killiany atlas regions.

To view the impact of the *ELOVL7* SE event in the brain, we analyzed the associations between the genotype-imputed PSI (${{{\hat{\mathrm \Psi }}}}$) and brain volumes from UKB. We found that the SE contributed to changes of gray matter volumes in multiple regions involved in processing auditory and visual information, such as the left and right Heschl’s gyrus and left occipital cortex (Fig. 4 and Supplementary Table S7). This result shows that the *ELOVL7* SE may impact specific brain regions, including the visual cortex which plays a role in alcohol addiction [64, 65].

## Discussion

The primary conclusion of the current study is that mRNA alternative splicing, specifically exon skipping, has a causal effect on AUD susceptibility. This conclusion is supported by the identification and replication of six exon skipping events; one was further substantiated in GWAS with diverse populations and additional AUD-related traits. Furthermore, some differentially expressed genes downstream of the identified events are known to be alcohol-responsive and associated with immunological and neurological pathways, providing additional evidence that AUD shares a genetic basis with immune and neural diseases. This knowledge advances our understanding of the contribution of RNA splicing to the genetic risk for AUD. In addition, our workflow can be a framework for splicing studies in the genetics of other complex diseases.

To date, the aim of most genomic or transcriptomic-scale studies of splicing in AUD has been to reveal how alcohol consumption affects splicing [12, 66]. Multiple studies have indicated that specific RNA splicing events are important in the brain and in neurological disorders [2, 3]. However, our understanding of the causal role of splicing on the susceptibility of AUD is limited. Therefore, we implemented a Mendelian randomization (MR)-based strategy to systematically assess the causality of splicing events in AUD. MR typically utilizes a modeling approach to map genetic variants to the molecular trait of gene expression, such as PrediXcan, TWAS, or SMR [15–17]. The purpose of the modeling is not to simply predict the molecular trait; rather, it is to determine the extent to which genetic variants can explain the molecular trait. In this study, we adapted MR from a gene expression-centric approach to accommodate RNA splicing analysis.

We established new splicing models to use with classic MR in identifying the specific splicing events instead of conducting sQTL analysis together with commonly used summary-based approaches as used in previous studies [13, 18–20]. Our strategy provides greater precision and specificity in terms of selecting the explanatory SNVs and dissecting the genetically determined component of RNA splicing. On one hand, summary-based studies infer a causal splicing event by co-localizing the SNV (Z)-splicing (X) association *β*_zx_ and the SNV (Z)-trait (Y) association *β*_zy_. In such approaches, the explanatory SNVs for splicing outcome are not identified, and the genetic component of the splicing outcome cannot be directly assessed. As a result, a challenge arises in evaluating the true validity of the model. In this regard, models established herein are verifiable directly through either heritability analysis or an independent RNA-seq dataset. On the other hand, identifying the SNVs responsible for alternative splicing events that impact disease susceptibility enables prediction of the disease risk based on individual genotyping information. Therefore, these models described herein facilitate future studies on personalized health care including AUD.

The adapted MR approach enabled us to identify six exon skipping events that impact AUD susceptibility. Interestingly, four of the five exons in protein coding genes are in the untranslated regions and two of these changed the expression of their host genes (*ELOVL7* and *NSUN4*), suggesting that they may be involved in post-transcriptional regulation. Of note, *ELOVL7*, was previously identified as down-regulated in prefrontal cortex in individuals with alcohol dependence [52]. *DRC1* encodes a critical component involved in regulating ciliary dynein motors that are targeted by alcohol-induced ciliary dysfunction [54]. *NSUN4* and *SRRM2* are both involved in neurological disorders [50, 51]; in particular, *SRRM2*, a splicing factor, regulates ethanol-cue-induced memory in flies [67]. Alcohol downregulates *TBC1D5*, which contributes to alcoholic liver disease as well as to Parkinson’s disease and Alzheimer’s disease [68]. Additionally, *LINC00665* is an emerging cancer biomarker, including in glioma and alcohol-related cancers (e.g., breast cancer and liver cancer) [69–71].

Additionally, of the 970 genes that are potentially regulated by at least one of the five analyzable skipped exons, 197 (20%, Supplementary Table S4) were responsive to alcohol in a cell culture study [57]. Among these genes, 20 have also been identified as alcohol-responsive in human and animal studies, further increasing their relevance to AUD. Together, these findings indicate that expression changes of these 197 genes are not simply an effect of alcohol intake, but rather, the expression of these genes contribute to the genetic basis of AUD. Two additional genes, *OAS3* (2’-5’-Oligoadenylate Synthetase 3) and *OXTR* (Oxytocin Receptor) show evidence in the NHGRI GWAS catalog for association with alcohol consumption and dependence, respectively. OAS3 is an interferon (IFN-*α*/*β* or *γ*)-induced, dsRNA-activated oligoadenylate synthase that plays a critical role in cellular antiviral response. OXTR is a G-protein coupled receptor for oxytocin, which is known to play a role in neuropsychiatric disorders, including alcohol and drug addiction [72, 73]. Moreover, we found that *Hsp70/HSPA6* was differentially expressed as a result of genetic variant-induced splicing changes in any of the three skipped exons in *ELOVL7, LINC00665*, and *NSUN4*. HSPA6 is a splicing factor found to be significantly upregulated upon alcohol intake in multiple brain regions, which suggests it may contribute to mis-splicing in the brain transcriptome [12]. Thus, although the functional roles of these genes in AUD have not been well studied, our findings provide evidence that they not only exhibit alcohol-induced effects, but may also contribute to the risk for AUD. The genes identified in our analysis that have not been described in earlier studies might also prove to be important for AUD risk.

Pathways that were enriched by the differentially expressed genes downstream of one or more of the causal splicing events include neural developmental pathways such as neurogenesis and gliogenesis, as well as neuropathological pathways such as Parkinson’s and Alzheimer’s diseases. Interestingly, each of the causal splicing events implicated the epithelial-mesenchymal transition (EMT) pathway, which underlies many fundamental biological processes, including neural tube formation and cancer metastasis [74]. The host gene *LINC00665* is known to regulate EMT in cancer [71], and alcohol stimulates the EMT program in cancer cells, which leads to cancer progression [75]. These data further support the overlap of AUD with neurodegenerative disease [76], as well as a role of splicing in the development of alcohol-related cancers.

Our findings indicate that type 1 interferon (IFN-*α*/*β*) signaling, along with the type 2 interferon (IFN-*γ*) pathway, are regulated by alternative splicing in AUD. For each of the five exon skipping events, their respective downstream genes were enriched in IFN-*α*/*β*/*γ* signaling pathways. The IFN-*α*/*β* pathway was found to be affected by alcohol in a previous cell culture study [57]. In addition, our findings provide further evidence for the relevance of neuroimmune pathways to AUD, including the TNF, NF-κB, IL6-JAK-STAT3, IL2-STAT5, and NOD-like receptor (NLR) signaling pathways, as well as T cell activation and differentiation, because they are regulated by one or more of these five splicing events. These neuroimmune pathways have previously been shown to be responsive to alcohol [57, 77]. Our results also showed that the exon skipping events in *ELOVL7, LINC00665*, *NSUN4*, and *SRRM2* implicate the complement cascade, which is part of the innate immune system. While the complement cascade is known to be involved in alcoholic liver disease, it also participates in neurodevelopment and protects the central nerve system from inflammation [78]. Taken together, our findings further support the relevance of inflammatory cytokine-induced immune response to AUD.

We observed that the *ELOVL7* exon skipping event exhibits greater significance for the association with the problematic alcohol use traits of *alcohol dependence*, *AUD*, and *AUDIT-P* (Fig. 3G–I), compared to the alcohol consumption trait of *drinks per week* (*DrnkWk*, Fig. 3J). A potential explanation for this finding is provided by a previous GWAS meta-analysis demonstrated that *drinks per week* differs from the other three AUD traits, having only a mild or moderate genetic correlation with them [11]. Together, these results provide high confidence for the role of *ELOVL7* in risk of AUD.

Moreover, the occipital cortex (left hemisphere, including the left primary and secondary visual cortices, BA 17 and 19, respectively) that is potentially impacted by the *ELOVL7* splicing event, is one of the main brain areas of neurological alterations induced by alcohol intake [79]. Furthermore, the occipital cortex is activated by drug cues including alcohol, as functional MRI signaled significant alcohol or drug-elicited activity in the left BA 19 and BA 17 [65]. In a recent study, AUD was found to be associated with change of the alpha oscillatory activity in the occipital cortex, indicating that the visual cortex plays a role in alcohol addiction [64]. In addition, the auditory cortex, i.e., Heschl’s gyrus, was impacted by the *ELOVL7* splicing event on both hemispheres. This area indeed exhibits alcohol-induced changes in brain functional connectivity studies [79, 80]; however, no studies to date have indicated its role in the development of alcohol addiction. Thus, although our observation here does not imply a causal role for these brain regions, it provides additional relevance of these cortical structures in alcohol use disorder.

One limitation of the current study is that, since it was designed to answer the general question whether RNA splicing impacts the genetics of AUD, it cannot answer cell type-specific questions. Our study design used bulk RNA-seq data because the statistical genetics analysis required the power of large-scale cohorts of samples with known genotypes, AUD-related phenotypes, and transcript-level quantifications. In addition, splicing analysis requires sufficient read depth that is not currently available in single-cell RNA sequencing data. Moreover, the relatively small sample size such as COGA and OZ-ALC may limit the power in discovering significant splicing events. Another limitation is that causality cannot be directly verified because of the challenges in experimentally modeling complex traits such as AUD, as cell culture studies and animal models cannot completely represent the human disease system. Nevertheless, a major strength of our study is that the use of the MR methodology, including the GSMR, leverages high power in causality inference from large-scale datasets. This method enables identification of causal splicing events, which provides new information on the role of RNA splicing in AUD risk. The new information regarding the downstream genes and pathways implicated by the splicing events provides evidence of causality for findings from previous GWAS and differential gene expression studies, as well as sheds new insights in the molecular mechanisms contributing to AUD. For example, targeting an alternatively spliced exon identified herein an experimental model system relevant to AUD could provide further verification. Together, our results advance the field of AUD research and our method provides a framework for studying RNA splicing in complex genetic diseases.

### Supplementary information

Supplementary Information

Supplementary Figure S1

Supplementary Figure S2

Supplementary Figure S3

Supplementary Table S1

Supplementary Table S2

Supplementary Table S3

Supplementary Table S4

Supplementary Table S5

Supplementary Table S6

Supplementary Table S7

## Data Availability

The datasets and tools used in the current study are available from the following sources. RNA-seq in DLPFC and genotypes from CMC: https://www.synapse.org/#!Synapse:syn2759792/wiki/69613. RNA-seq in PFC and genotypes of samples from NSWBTRC: https://www.sydney.edu.au/medicine-health/schools/school-of-medical-sciences/nsw-brain-tissue-resource-centre.html. Genotypes and phenotypes from COGA: https://www.niaaa.nih.gov/research/major-initiatives/collaborative-studies-genetics-alcoholism-coga-study. Genotypes and phenotype from OZ-ALC: https://www.ncbi.nlm.nih.gov/projects/gap/cgi-bin/study.cgi?study_id=phs000181.v1.p1. GTEx database (V8): https://gtexportal.org/home. GWAS summary statistics of PGC: https://www.med.unc.edu/pgc. GWAS summary statistics of GSCAN: https://genome.psych.umn.edu/index.php/GSCAN. GWAS summary statistics of MVP: https://www.research.va.gov/mvp. GWAS summary statistics and MRI data of UKB: https://www.ukbiobank.ac.uk/. This study did not generate code.
