# Supplementary material for: RNA alternative splicing impacts the risk for alcohol use disorder
Source: Mol Psychiatry. 2023 May 23;28(7):2922–33. doi: 10.1038/s41380-023-02111-1 (PMC10615768; doi:10.1038/s41380-023-02111-1)
Supplement: Supplementary file 1 — Supplementary Information [file 41380_2023_2111_MOESM1_ESM.pdf]

Supplementary information

Supplementary Figure S1. Proportion of PSI explained by the *cis*-regulatory elastic-net (EN) model (PDF). Legends are in the file.
Supplementary Figure S2. Sample stratification of the six causal splicing events based on *cis*-regulated PSI (PDF). Legends are in the file.
Supplementary Figure S3. Number of downstream DE genes for each of the identified splicing events (PDF). Legends are in the file.
Supplementary Table S1. Summary of 27 candidate exon skipping events for AUD risk (XLSX).
Supplementary Table S2. Summary of 51 genetic variants most explanatory to the final six skipped exons (XLSX).
Supplementary Table S3. Full list of downstream differentially expressed genes for each skipped exons (XLSX).
Supplementary Table S4. Summary of alcohol-response genes studied in cell culture, human and rat brains, and previous GWAS (XLSX).
Supplementary Table S5. Full result of GO enrichment analysis (XLSX).
Supplementary Table S6. Full result of GSEA analysis including three pathway databases GO, KEGG, and MSigDB Hallmark (XLSX).
Supplementary Table S7. Summary of associations between the *ELOVL7* exon skipping event with grey matter volumes in multiple brain regions (XLSX).
