# Supplementary material for: RNA alternative splicing impacts the risk for alcohol use disorder
Source: Mol Psychiatry. 2023 May 23;28(7):2922–33. doi: 10.1038/s41380-023-02111-1 (PMC10615768; doi:10.1038/s41380-023-02111-1)
Supplement: Supplementary file 2 — Supplementary Figure S1 [file 41380_2023_2111_MOESM2_ESM.pdf]

Figure S1

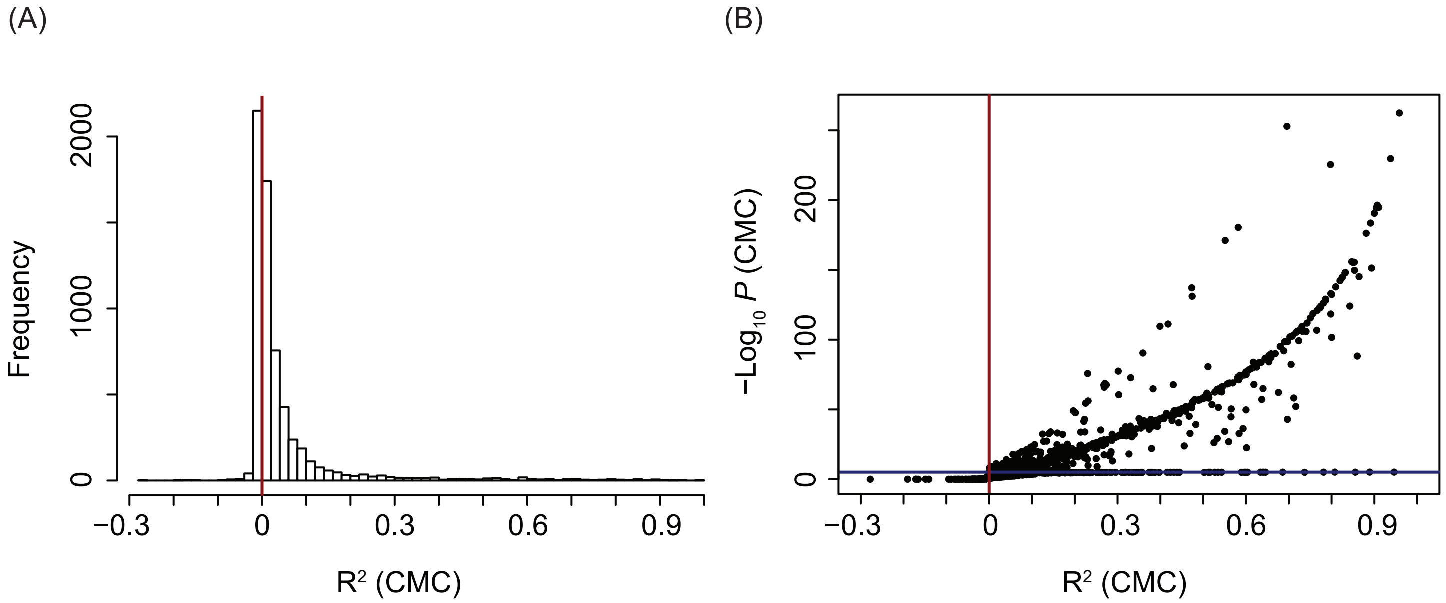

(A)
Frequency
0
1000
2000
−0.3
0
0.3
0.6
0.9
R2 (CMC)
(B)
−Log10 P (CMC)
0
100
200
−0.3
0
0.3
0.6
0.9
R2 (CMC)

Figure S1. Proportion of PSI explained by the cis-regulatory elastic-net (EN) model.

(**A**) Histogram of model-explained proportions, R^2 (x-axis), of the CMC RNA-seq PSI for 6,284 SE. Red line, $R^2 = 0$. Zero or negative R^2 values indicate the PSI is not explainable by the genetic variants. For 64.8% of the SE, the model-explained $R^2 > 0$.
(**B**) Plot shows the model significance ($-\log_{10} P$, y-axis) versus the R^2 (x-axis). Red line, $R^2 = 0$; blue line, $P = 7.96\times10^{-6}$ (Bonferroni *p* cutoff). Dots with $R^2 > 0$ and $P < 7.96\times10^{-6}$ correspond to highly cis-regulated events (n = 1,093 SE).
