# Supplementary material for: RNA alternative splicing impacts the risk for alcohol use disorder
Source: Mol Psychiatry. 2023 May 23;28(7):2922–33. doi: 10.1038/s41380-023-02111-1 (PMC10615768; doi:10.1038/s41380-023-02111-1)
Supplement: Supplementary file 3 — Supplementary Figure S2 [file 41380_2023_2111_MOESM3_ESM.pdf]

Figure S2

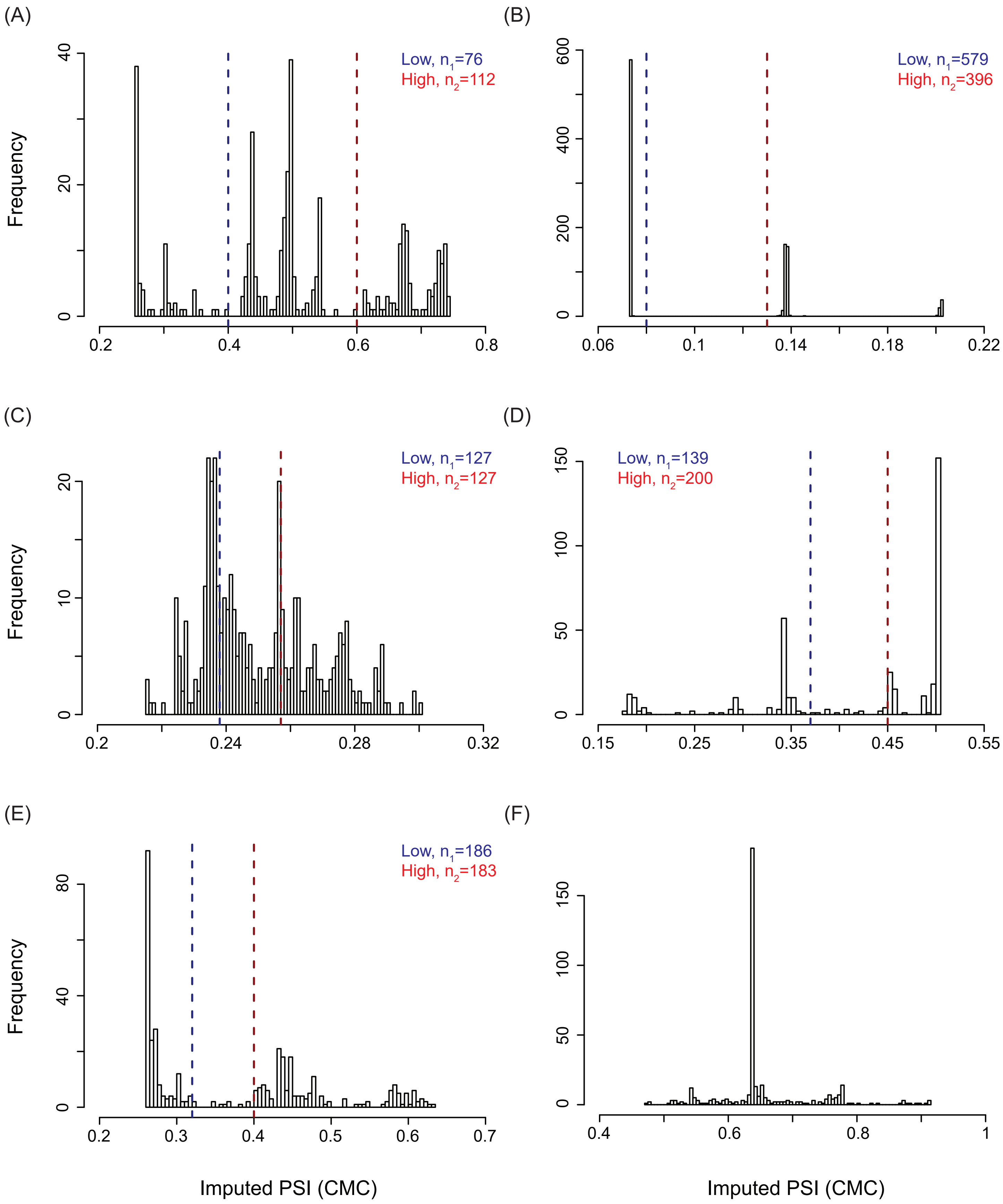

(A)
Low, n_1=76
High, n_2=112
Frequency
0
20
40
0.2
0.4
0.6
0.8
(B)
Low, n_1=579
High, n_2=396
0
200
400
600
0.06
0.1
0.14
0.18
0.22
(C)
Low, n_1=127
High, n_2=127
Frequency
0
10
20
0.2
0.24
0.28
0.32
(D)
Low, n_1=139
High, n_2=200
0
50
100
150
0.15
0.25
0.35
0.45
0.55
(E)
Low, n_1=186
High, n_2=183
Frequency
0
40
80
0.2
0.3
0.4
0.5
0.6
0.7
Imputed PSI (CMC)
(F)
0
50
100
150
0.4
0.6
0.8
1
Imputed PSI (CMC)

Figure S2. Sample stratification of the six causal splicing events based on cis-regulated PSI.

CMC samples with genetically imputed PSI values less than the level marked by the blue dashed line (n_1) were labeled as low, and those greater than the level marked by the red dashed line (n_2) were labeled as high. Intervening samples (n_0) with PSI values between the high and low were unused.
(**A**) ENSE00002438745 in *LINC00665*. $n_1 = 76$, $n_2 = 112$, $n_0 = 192$.
(**B**) ENSE00001875548 in *NSUN4*. $n_1 = 579$, $n_2 = 396$, $n_0 = 0$.
(**C**) ENSE00002674786 in *SRRM2*. $n_1 = 127$, $n_2 = 127$, $n_0 = 126$.
(**D**) ENSE00002079807 in *ELOVL7*. $n_1 = 139$, $n_2 = 200$, $n_0 = 41$.
(**E**) ENSE00003572542 in *DRC1*. $n_1 = 186$, $n_2 = 183$, $n_0 = 11$.
(**F**) ENSE00001693995 in *TBC1D5*. Samples on the high and low ends are not of sufficient numbers.
