# Supplementary material for: RNA alternative splicing impacts the risk for alcohol use disorder
Source: Mol Psychiatry. 2023 May 23;28(7):2922–33. doi: 10.1038/s41380-023-02111-1 (PMC10615768; doi:10.1038/s41380-023-02111-1)
Supplement: Supplementary file 4 — Supplementary Figure S3 [file 41380_2023_2111_MOESM4_ESM.pdf]

Figure S3

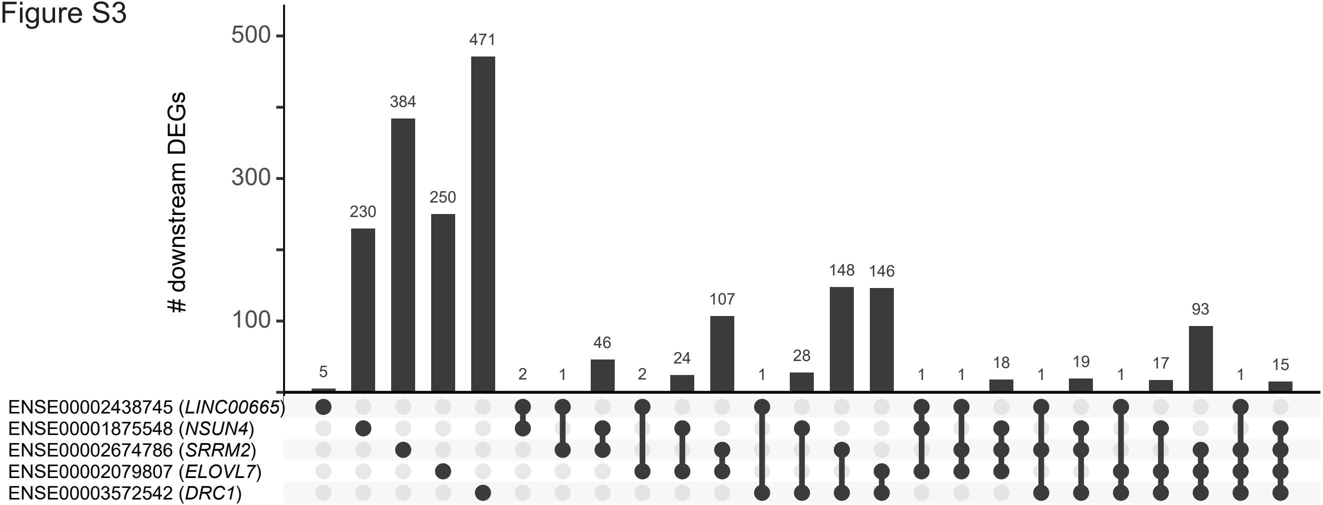

500
300
100
downstream DEGs
5
230
384
250
471
2
1
46
2
24
107
1
28
148
146
1
1
18
1
19
1
17
93
1
15
ENSE00002438745 (*LINC00665*)
ENSE00001875548 (*NSUN4*)
ENSE00002674786 (*SRRM2*)
ENSE00002079807 (*ELOVL7*)
ENSE00003572542 (*DRC1*)

Figure S3. Number of downstream DE genes for each of the causal splicing events.

Five events corresponding to 1-5 in **Table 1** were listed at the bottom of the UpSet plot. Numbers of their respective downstream genes, as well as overlapping genes between or among these sets of downstream genes, were shown by the bars. Only the intersections with non-zero number of overlapping genes were shown.
